# Use of the neuroform atlas for stenting of intracranial atherosclerotic disease: Clinical and angiographic outcomes

**DOI:** 10.1177/15910199231195134

**Published:** 2023-10-10

**Authors:** Yosef Ellenbogen, Eef J Hendriks, Spyros Karadimas, Sean O’Reilly, Zeev Itsekzon Hayosh, Rabab Alshahrani, Ronit Agid, Joanna Schaafsma, Timo Krings, Patrick Nicholson

**Affiliations:** 1 Division of Neurosurgery, Department of Surgery, University Health Network, Toronto, Ontario, Canada; 2 Division of Neuroradiology, Joint Department of Medical Imaging, University Health Network, Toronto, Ontario, Canada

**Keywords:** Neuroform atlas stent, intracranial atherosclerosis, intracranial stent, angioplasty

## Abstract

**Background:**

Intracranial atherosclerotic disease (ICAD) is a potential cause of ischemic stroke. Treatment of ICAD can include intracranial stenting. There are specifically designed stents for this use-case; however, less is known about the off-label use of the Neuroform Atlas stent. In this study, we describe the outcomes of the Neuroform Atlas stent for treatment of ICAD.

**Methods:**

Adult patients with symptomatic ICAD failing best medical treatment undergoing elective intracranial stenting using the Neuroform Atlas stent between November 2018 and March 2021 were included. Patient demographics, procedure-related details and clinical and imaging outcomes were analyzed.

**Results:**

Eighteen patients met the inclusion criteria, with a mean follow-up duration of 9.6 ± 6.8 (standard deviation) months. There were two procedure-related mortalities (one massive intracranial hemorrhage and one groin site complication with sepsis). Fifteen patients were alive at the 6-month follow-up, all with satisfactory stent patency on follow-up imaging without any new ischemic events. Modified Rankin Scale at latest follow-up was 1.9 (interquartile range 5).

**Conclusion:**

In this single-center consecutive series, intracranial stenting with the Neuroform Atlas stent was a safe and effective treatment for symptomatic ICAD patients failing best medical management.

## Introduction

Ischemic stroke is the second leading cause of death globally after ischemic heart disease;^1^ 10–20% of all ischemic strokes are caused by intracranial atherosclerosis,^2^ while intracranial plaques and stenoses are found in 31–62% of patients with a history of ischemic strokes.^3,4^ Higher rates of intracranial atherosclerotic disease (ICAD) are found in Asian patients,^1,5^ as well as in the Afro-American and Hispanic population. Risk factors associated with intracranial atherosclerosis include hypertension, smoking, diabetes and hyperlipidemia.^6–10^ The potential mechanisms for intracranial atherosclerosis-related strokes are threefold: plaque rupture with arterio-arterial embolism,^11^ hypoperfusion with hemodynamic compromise of the watershed areas,^12^ and perforator artery ostial occlusion by the plaque.^13^

Symptomatic intracranial atherosclerosis is typically managed with medical therapy, usually with antiplatelet agents and risk factor modification. Stenting has been performed in case of recurrent strokes after failing best medical management. Recent studies, such as the prospective WEAVE trial, have demonstrated that stenting of ICAD can be performed with a favourable safety profile in selected patients.^14^ The follow-up WOVEN trial study showed an 8.5% risk of stroke/death at 1 year.^15^ These studies used the Wingspan Stent (Stryker Neurovascular, Fremont, CA, USA), one of the most commonly used and approved stent systems to treat ICAD. Notably, the Wingspan Stent requires the use of an exchange-wire system,^14,16^ whereas the newer, low-profile Neuroform Atlas stent (Stryker Neurovascular, Fremont, CA, USA) holds the advantage of not requiring an intracranial wire exchange manoeuvre.^17,18^ Specifically, it allows for a lower profile delivery of the stent. Prior stents require a 0.027-inch inner diameter delivery microcatheter, whereas the Neuroform Atlas accommodates 0.0165-inch inner diameter microcatheters, such as an SL-10 or Echelon 10. For the past 3 years, in our institution, we therefore have preferentially used a Neuroform Atlas for treatment of ICAD patients. In the present study, we examine the clinical, technical and radiographic outcomes of 18 consecutive patients who underwent balloon angioplasty and stenting of symptomatic ICAD using the Neuroform Atlas system after failed best medical therapy, with at least 3 months of follow-up.

## Methods

This retrospective cohort study analyzes patients who underwent stenting of intracranial atherosclerosis using the Neuroform Atlas stent. Consecutive patients were included between November 2018 and March 2021. Ethics approval was obtained from our institutional review board (CAPCR-ID: 21-6053.0). All adult patients with symptomatic ICAD who, after failed best medical therapy, underwent elective treatment with balloon angioplasty and stenting using the Atlas stent system. Exclusion criteria consisted of use of the Neuroform Atlas for stenting an underlying stenosis in the context of acute stroke (i.e., as a ‘rescue’ therapy after endovascular thrombectomy for acute large vessel occlusion secondary to underlying ICAD).

### Data collection

For all cases, clinical, procedural and angiographic data were collected. This included demographic data, presenting symptoms, location of intracranial stenosis, endovascular approach, materials used, use of angioplasty before or after stenting and antiplatelet/anticoagulant regimen. Additionally, all intra- and peri-procedural events were recorded during the hospitalization. Outcome data was assessed using the modified Rankin Scale (mRS) at the time of discharge, and at latest follow-up. Additionally, angiographic outcomes were collected as well. All patients were followed in the neurovascular clinic as well as by a stroke neurologist. Complications were divided into major and minor complications. A major complication was defined as being directly related to the procedure with symptoms persisting for longer than 7 days from the event.

### Procedure technique

**Figure 1.**
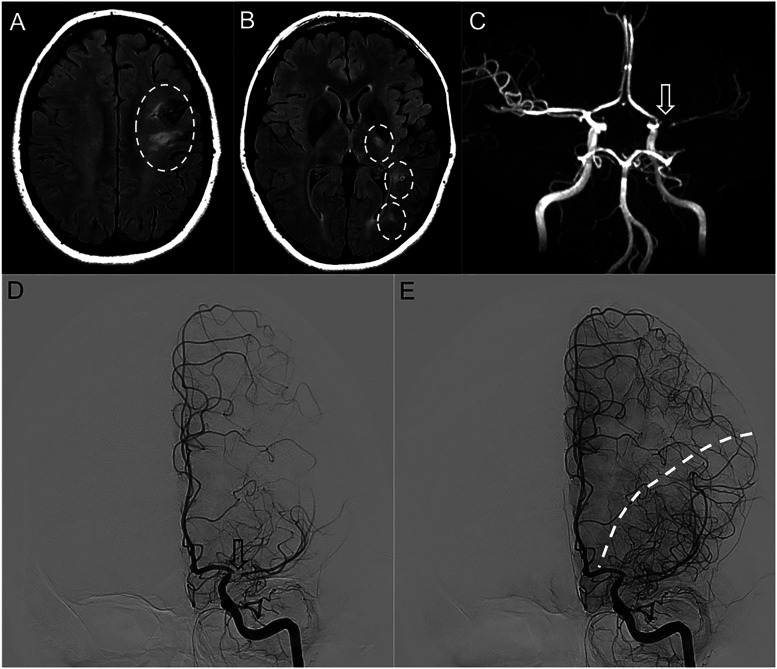
Axial T2-FLAIR images demonstrated areas of hyperintensity and gliosis in the left frontal lobe (a), left corticospinal tract and left optic radiation (b). MRA-TOF imaging (c) demonstrated high-degree stenosis of the left proximal M1 segment (arrow). Digital subtraction angiography (DSA) demonstrating left ICA injections in the early arterial (d) and late arterial (e) phases, confirming the steno-occlusive segment of the proximal left M1 segment (arrow). Late arterial phase showed a shift of the watershed towards the 3 o’clock position (dashed line), with extensive collaterals from the left ACA.

**Figure 2.**
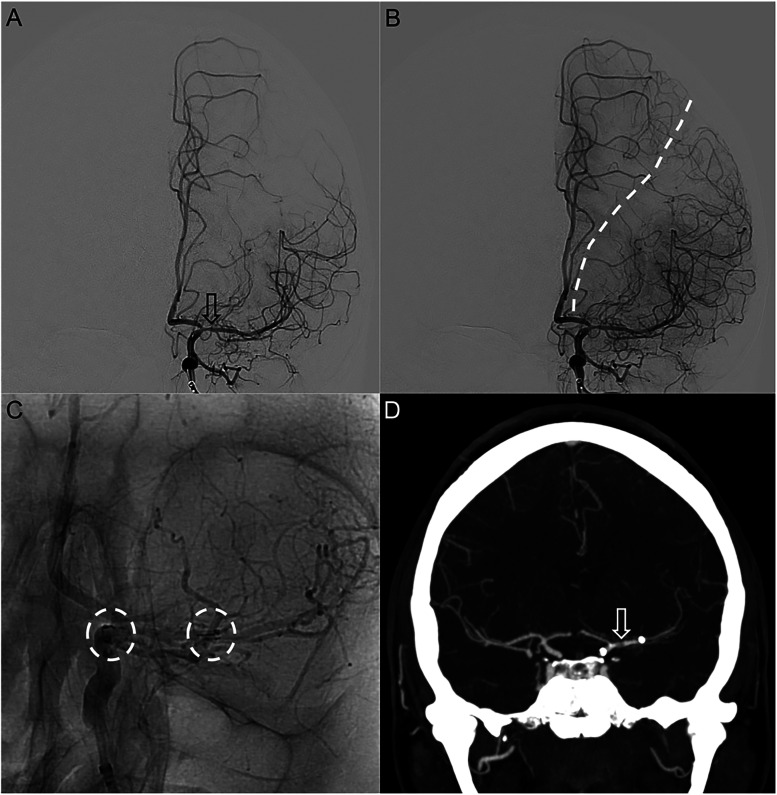
ICA injection of early arterial phase showing significant improvement in caliber of the proximal M1 (arrow) post Neuroform Atlas stenting (a). Late arterial phase demonstrating shift of the watershed towards the 1 o’clock position (dashed line) (b). Additional unsubtracted injection of the left ICA (c) showed the proximal and distal landing zones of the stent, from the distal ICA into the left mid M1 segment. Follow-up CTA after 3 months (d) confirmed remaining patency of the left MCA stent (arrow).

Patients were preloaded with aspirin and either clopidogrel or ticagrelor (as per individual operator preference) for 5 days before the procedure. After 6 months, the majority of the patients were transitioned to a single antiplatelet agent per institutional guidelines and in consultation with a multi-disciplinary team including a stroke neurologist. Three patients also met criteria for being on anticoagulation (two on warfarin and one on apixaban) and, therefore, only a single antiplatelet agent was used to minimize risk of bleeding complications. Platelet testing was not routinely performed. Treatment was performed 38 days (mean, standard deviation [SD] 83.9) following the qualifying event. All procedures were done under general anesthesia in a biplanar angiography suite. Following placement of a guide-catheter in the proximal ICA (usually either a 6-Fr Benchmark or an 8-Fr NeuronMax; Penumbra Inc, CA, USA) and a control angiogram, the symptomatic stenosis was crossed with an angioplasty balloon over a 300 cm 0.014″ wire (usually either a regular Synchro or Synchro Support; Stryker Neurovascular, Fremont, CA, USA). The balloon was sized to the affected vessel, usually a 2 × 6 mm MiniTrek coronary over-the-wire balloon (Abbott, Chicago, IL, USA). After slow submaximal angioplasty up to 4 atmosphere (atm) with an inflation speed of 1 atm per 20–40 s and confirmatory angiography, the balloon was rapidly exchanged for a microcatheter (most often an SL-10), which was then advanced across the now-dilated stenosis. The SL-10 was navigated in the distal vessel to support the stent, usually into the M2 MCA segment in case of an M1 stenosis. After introducing and backflushing the stent, it was deployed across the stenosis by a sole unsheathing technique. Control angiogram was performed to assess the need for post angioplasty. We occasionally perform a VasoCT to assess the stent following deployment. In case of early venous drainage in the treated territory or signs of hyperperfusion (i.e., ‘hypervascular blush’) on control angiogram, the systolic BP was medically decreased routinely by 30 mmHg for 24 to prevent a hyperperfusion syndrome. Postprocedural patients were admitted for at least 24 h to a medium or intensive care bed for strict monitoring of blood pressure and neuro vitals. Dual antiplatelet therapy was continued for at least 3 months. Conversion to single antiplatelet therapy was decided after a 3-month CTA to assess for stent patency. An MRI in the peri-procedural period was performed based on clinical indication. A representative case (paitent 14) is illustrated in Figures 1 and 2.

## Results

### Baseline characteristics

We identified 18 patients who met inclusion criteria (Tables 1 and 2). The mean age at time of stenting was 71 years (range 36–86). The cohort consisted of 10 male and 8 female patients. The types of stroke encountered in our group were hemodynamic in 10 cases (55.6%), mixed hemodynamic/embolic in 2 (11.1%), mixed hemodynamic/perforator in 2 (11.1%) and perforator only in 1 (5.6%). Ethnicity was Caucasian in 10 patients (66.7%), Asian in 4 (22.2%), Hispanic in 3 (16.7%) and Afro-American in 1 (5.6%). Risk factors for ICAD were hyperlipidemia in 16 (88.9%), hypertension in 14 (77.8%), diabetes in 9 (50%) and smoking in 1 (5.6%). The majority of the target vessel was in the anterior circulation with seven in the proximal MCA (right 1, left 5) and nine in the intracranial ICA (right 6, left 3). Three patients underwent stenting of the basilar artery. Balloon angioplasty prior to stenting was indicated in 17 (94.4%) cases. We performed post-stenting angioplasty in a single case. The imaging was assessed by a fellowship-trained neuroradiologist.

**Table 1.** Summary of patient cohort.

	Total (*N* = 18)
**Age (years)**	70.6 ± 12.1
**Follow-up duration (months)**	9.6 ± 6.8
**Sex**	
Male	10 (56)
Female	8 (44)
**Ethnicity**	
Caucasian	10 (55)
Asian	4 (22)
Hispanic	3 (17)
Afro-American	1 (6)
**Risk factors**	
Hyperlipidemia	16 (89)
Hypertension	14 (78)
Diabetes	9 (50)
Smoking	1 (6)
**Type of stroke**	
Hemodynamic	10 (56)
Mixed HD/Embolic	2 (11)
Mixed HD/Perforator	2 (11)
Perforator	1 (6)
**Location of stent**	
Right MCA	1 (6)
Left MCA	5 (28)
Right ICA	6 (33)
Left ICA	3 (17)
Basilar	3 (17)
**Angioplasty before stent**	17 (94)
**Angioplasty after stent**	1 (6)
**Antiplatelet**	
Aspirin and Clopidogrel	15 (83)
Single agent	3 (17)
**Anticoagulant**	
Warfarin	2 (11)
Apixaban	1 (6)

HD: hemodynamic stroke.

**Table 2.** Patient cohort.

Patient No.	Age	Sex	Location of stenosis	Angioplasty before stent	Angioplasty after stent	Antiplatelet	Anticoagulant	Complication	mRS at latest follow-up	Follow-up duration (months)*
1	76	Female	Right ICA	Yes	No	ASA & Plavix	No	No	6	N/A**
2	69	Male	Right ICA	Yes	No	ASA & Plavix	No	No	0	29.4
3	82	Female	Left ICA	Yes	No	ASA & Plavix	No	No	5	7.9
4	69	Female	Basilar	Yes	No	ASA & Plavix	No	GI bleed	5	13.7
5	60	Female	Basilar	No	Yes	ASA & Plavix	No	No	0	9.5
6	67	Female	Right ICA	Yes	No	ASA & Plavix	No	No	0	10.7
7	63	Female	Right ICA	Yes	No	ASA & Plavix	No	CFA thrombus	6	N/A
8	77	Male	Basilar	Yes	No	ASA & Plavix	No	No	1	12.5
9	86	Male	Left MCA	Yes	No	Plavix	Warfarin	Hemorrhagic transformation (PH2)	6	N/A
10	86	Male	Left MCA	Yes	No	Ticagrelor	Apixaban	CFA pseudoaneurysm	3	11.8
11	68	Male	Left MCA	Yes	No	ASA & Plavix	No	No	2	5.6
12	73	Male	Left ICA	Yes	No	ASA & Plavix	No	No	1	5.7
13	76	Female	Right ICA	Yes	No	ASA & Plavix	No	No	6	N/A**
14	36	Female	Left MCA	Yes	No	ASA & Plavix	No	No	1	3.0
15	61	Male	Left ICA	Yes	No	ASA & Plavix	No	No	0	4.5
16	78	Male	Left MCA	Yes	No	ASA	Warfarin	No	0	3.5
17	61	Male	Right MCA	Yes	No	ASA & Plavix	No	No	0	5.8
18	83	Male	Right ICA	Yes	No	ASA & Plavix	No	No	0	5.8

*Radiographic follow-up.

**Patient died prior to 6-month follow-up due to unrelated disease.

### Outcomes

Mean duration of follow-up was 9.6 ± 6.8 months (range 2–881 days; Table 3). There were no patients lost to follow-up, and 15 patients had follow-up of ≥6 months. There were two mortalities prior to the 30-day follow-up and one patient who died after the 90-day timepoint but prior to their 6-month follow-up due to unrelated illness. In the short-term follow-up, all stents were patent without any early in-stent occlusion or restenosis. In the cohort of patients with ≥6-month follow-up, all 15 demonstrated satisfactory appearances of the stent without any restenosis and with adequate distal flow. The mean mRS at latest follow-up was 1.9 (interquartile range [IQR] 5). At the latest follow-up, none of the patients had developed recurrent transient ischemic attacks (TIAs)/strokes or new neurologic deficits compared to their pre-operative assessment.

**Table 3.** Outcomes.

Follow-up imaging completed >6 months	15 (83)
Stent patent at latest follow-up	15 (100)
mRS at latest follow-up*	1.9 (IQR 5)
**Mortality**	
30 days	2 (11)
90 days (total)	2 (11)
**Major complication**	
Hemorrhagic transformation (PH2)	1 (6)
**Minor complication**	
Access complications	2 (11)
GI bleed	1 (6)

SD: standard deviation; IQR: interquartile range.

*MRS available in 16 patients.

### Adverse events

There were no adverse intra-procedural events. All stents were patent on the last intra-procedural angiographic run, and there were no cases of intra-procedural stent thrombosis. In no cases did we have to add eptifibatide due to unexpected platelet aggregation. There were two 30-day mortalities, one due to a groin site complication with sepsis and one due to a delayed intracranial hemorrhage at day 2 that was presumed to be related hyperperfusion injury (patient no. 7 and 9, respectively). Additionally, one patient experienced a femoral artery pseudoaneurysm that was treated with thrombin, which was classified as a minor adverse event. One patient developed an upper gastrointestinal bleed within 12 months of the stenting, which was successfully managed with a blood transfusion, temporarily holding the antiplatelet agents (<7 days), and initiation of a proton pump inhibitor. This patient did not develop new strokes and their stent remained patent.

### Mortalities

1. Patient no. 7 was a 64-year-old female with pre-existing severe peripheral vascular disease who underwent left ICA stenting for recurrent TIA and watershed infarcts of the left MCA territory. She developed a common femoral artery thrombus at the puncture site postoperatively that resulted in critical leg ischemia requiring open embolectomy and fasciotomy. She died of complications relating to sepsis and heart failure during the same admission.
2. Patient no. 9 was an 86-year-old male with a history of atrial fibrillation and chronic kidney disease on dialysis. He presented with fluctuating aphasia and was found to have critical left M1 stenosis with hypoperfusion of the left MCA territory. He was taking warfarin for management of his atrial fibrillation. Given this, he was initiated on a single antiplatelet agent, clopidogrel, prior to the procedure. There were no intra-procedural adverse events. However, within 4 h from the procedure, he was found to have a decreased level of consciousness with right-sided hemiparesis and CT showed evidence of hyperperfusion injury with contrast staining in the left basal ganglia. Despite supportive management and maintaining a systolic blood pressure less than 140 mmHg, this transitioned to a non-survivable massive intraparenchymal hemorrhage (PH-2).

## Discussion

Ischemic stroke is a debilitating condition that is one of the leading causes of death worldwide. In patients with ICAD, despite medical management, there remains a high risk of recurrent ischemic stroke. In a prospective study of 102 patients, 38.2% of patients experienced a new cerebrovascular event within 2 years (13.7% ischemic stroke and 24.5% TIA).^19^ In another prospective study among patients with ICAD causing ischemic stroke or TIA and treated medically, 8.8% has another stroke and 5.9% had a TIA within a year of follow-up.^20^

Several studies have evaluated the safety and efficacy of stenting with various stents. Most notably, the SAMMPRIS trial using the Wingspan stent demonstrated a high rate of 30-day death or stroke post stenting.^21^ This was attributed to stenting of patients who did not meet the Humanitarian Device Exemption on-label indications for stenting, including stenting patients who had not failed medical therapy. The WEAVE trial with the Wingspan stent subsequently showed lower than expected periprocedural stroke, bleed and death rate.^14^ Still, the main limitation of the Wingspan stent is the intracranial manoeuvre required to exchange the wire with a longer (300 cm, 0.014 inch) wire. This has been hypothesized to have resulted in the higher rate of ipsilateral cerebral hemorrhage seen in the SAMMPRIS trial.^16^

The Neuroform Atlas stent is the latest iteration of the Neuroform stent models. It is a self-expanding laser-cut nitinol stent that combines closed cells in the proximal end and open cells in the distal end to facilitate anchoring and wall apposition.^22^ In contrast to the Wingspan stent, the Neurofrom Atlas allows for stenting without requiring angioplasty as it is a self-expandable stent rather than balloon mounted. Its initial use has been focused on the treatment of stent-assisted coiling of wide-necked intracranial aneurysms.^17^ However, recently, its efficacy and safety in intracranial stenting has been demonstrated in several retrospective studies.^18,22–24^ For example, in a retrospective study of 10 patients with symptomatic high-grade intracranial stenosis, Buonomo et al. reported no new ischemic events at 3-month follow-up, and a statistically significant reduction in the stenotic segment of the vessel after stenting.^23^ In another study Yi et al., the use of the Neuroform Atlas stent was assessed as a rescue manoeuvre after failure of endovascular thrombectomy for large vessel occlusion. Their experience consisted of 31 patients with 90.3% having tolerable vessel patency at 14 days post stenting.^18^

In the present work, the characteristics and outcomes of 18 patients with symptomatic ICAD who underwent elective stenting with the Neuroform Atlas system are presented. Out of the 18, two died in the first 30 days after stenting, one of which was related to perioperative intracranial hemorrhage, and the other from pre-existing medical complications exacerbated by a groin site complication (CFA thrombus). Out of the remaining patients, 15 had follow-up past 6 months, and all 15 demonstrated stent patency with good distal flow on CTA. There were no recurrences of strokes or TIAs at the latest follow-up.

Most notably, there were no new incidences of stroke in the periprocedural or follow-up periods. This is an important point, as it is significantly less than the 14.7% seen in SAMMPRIS^23^ (30-day follow-up) and 2.6% in WEAVE^14^ (72-h periprocedural). To our knowledge, this is largest study to date assessing the use of Neuroform Atlas stent for the elective treatment of intracranial atherosclerosis. The technical and clinical outcomes in our series demonstrate good safety and efficacy of the system. All complications were related to either access or antiplatelet/anticoagulant use, rather than stent-related issues. Notably, we feel that the advantages mentioned such as the lack of the need for an exchange manoeuvre or regular post-stenting angioplasty make it an advantageous and versatile system. It is for this reason that it has now become our first-line choice for treatment of ICAD in both acute and elective situations.

This study suffers from certain limitations. Notably, as a single-center retrospective cohort of 18 patients, the results should be interpreted with caution due to inherent selection bias and relatively low numbers. We attempted to reduce selection bias by including all consecutive patients. Additionally, while the initial follow-up data is encouraging, long-term follow-up of its durability is necessary.

## Conclusion

Overall, this study demonstrates the viability of the Neuroform Atlas Stent System for safe and effective treatment of symptomatic intracranial atherosclerosis. Larger prospective cohorts are needed to thoroughly evaluate this stent for the treatment of intracranial atherosclerosis and validate the results of this retrospective analysis.
